# The Ultrafast and Accurate Mapping Algorithm FANSe3: Mapping a Human Whole-Genome Sequencing Dataset Within 30 Minutes

**DOI:** 10.1007/s43657-020-00008-5

**Published:** 2021-02-22

**Authors:** Gong Zhang, Yongjian Zhang, Jingjie Jin

**Affiliations:** 1 MOE Key Laboratory of Tumor Molecular Biology and Key Laboratory of Functional Protein Research of Guangdong Higher Education Institutes, Institute of Life and Health Engineering, College of Life Science and Technology, Jinan University, Guangzhou, 510632 China; 2 Chi-Biotech Co. Ltd., Shenzhen, 518000 China

**Keywords:** Next-generation sequencing, Mapping, Speed, Robustness

## Abstract

Aligning billions of reads generated by the next-generation sequencing (NGS) to reference sequences, termed “mapping”, is the time-consuming and computationally-intensive process in most NGS applications. A Fast, accurate and robust mapping algorithm is highly needed. Therefore, we developed the FANSe3 mapping algorithm, which can map a 30 × human whole-genome sequencing (WGS) dataset within 30 min, a 50 × human whole exome sequencing (WES) dataset within 30 s, and a typical mRNA-seq dataset within seconds in a single-server node without the need for any hardware acceleration feature. Like its predecessor FANSe2, the error rate of FANSe3 can be kept as low as 10^–9^ in most cases, this is more robust than the Burrows–Wheeler transform-based algorithms. Error allowance hardly affected the identification of a driver somatic mutation in clinically relevant WGS data and provided robust gene expression profiles regardless of the parameter settings and sequencer used. The novel algorithm, designed for high-performance cloud-computing after infrastructures, will break the bottleneck of speed and accuracy in NGS data analysis and promote NGS applications in various fields. The FANSe3 algorithm can be downloaded from the website: http://www.chi-biotech.com/fanse3/.

## Introduction

Next-generation sequencing (NGS) is a key cornerstone in precision medicine. With the rapid decrease in the experimental cost of NGS, human whole-genome sequencing (WGS) at 30× depth can be performed at the cost of $700, and an mRNA sequencing (RNA-seq) costs only $80. In contrast, the cost of NGS data analysis remains nearly unchanged. Mapping i.e., the alignment of millions of short reads to reference sequences, is the most computationally intensive step in NGS data analysis, and it requires a fast, accurate and robust mapping algorithm.

Burrows–Wheeler transform (BWT)-based mapping algorithms such as the Burrows–Wheeler alignment (BWA) and Bowtie tools are the most widely used algorithms in NGS applications owing to their great advantages in speed compared to that of normal seed-based algorithms. They can map a human WGS dataset within 1 day in a server node (Hung and Weng 2017). Hardware acceleration using GPU (Graphical Processing Unit) or FPGA (Field-Programmable Gate Array) further accelerates with BWT-based mapping by 2–7 times (Nogueira et al. 2016) and enables mapping of a human WGS dataset within 3 h in a single server node. However, due to the sequencing error and deviation "between reads and reference sequences, BWT-based algorithms generally lose accuracy when the error rate exceeds 2%. These algorithms lose numerous mappable reads in real-world tests at unpredictable rates (Schbath 2012; Ruffalo et al. 2011; Fonseca 2012; Homer et al. 2009), indicating their extreme dependence on sequencing quality and genetic background (Hung and Weng 2017). Whole exome sequencing (WES) for 57 patients with genetic diseases failed to detect any Human Gene Mutation Database-cataloged variant, showing an unacceptable high false-negative rate (Park 2015). Disease-causing mutations in 7 genes were undetected due to the inadequate coverage. Other failures are mainly caused by instability and inaccuracy of the algorithms, the concordance rate of single-nucleotide variants (SNVs) identified by five commonly used algorithms was only 26.8–57.4% (O'Rawe 2013).

To meet the accuracy and speed demands of precision medicine, we developed the FANSe series of mapping algorithms (Mai 2017; Xiao 2014; Zhang 2012). Unlike other algorithms, which generally prioritize speed, FANSe prioritizes accuracy. In WGS variant calling, experimental validation of 1994 sites revealed no false positives or negatives (Wu 2014). The RNA-seq mRNAs identified by FANSe2 could be perfectly validated by RT-PCR whereas those identified by Bowtie2 could not (Xiao 2014). This high accuracy made the FANSe algorithms optimal for the analysis of translating mRNA sequencing (RNC-seq) data, which is one of the key resource pillars in the Human Proteome Project (Zhong 2014) and it thus facilitates sensitive and accurate identification of proteins from shotgun mass spectrometry data (Chang 2014; Zhang et al. 2014; Xu 2015; Zhao 2017). Moreover, differentially expressed genes identified from FANSe2-mapped data were perfectly validated by RT-qPCR (Li 2017). The FANSe2splice algorithm, which is designed to map spliced reads to a genome based on the FANSe principle, in terms of experimental verifiability outperformed other mainstream spliced mappers, such as TopHat2 (Kim 2013), MapSplice2 (Wang 2010), HISAT2 (Kim et al. 2015), and STAR (Dobin 2013), and it can detect splice junctions from low-throughput semi-single-cell sequencing datasets (Mai 2017). Besides their high accuracy, the FANSe algorithms are highly error-tolerant and can be effectively applied to the meta-genome/meta-transcriptome studies, of various non-model organisms outperforming other algorithms (reviewed in Cao and Zhang 2017).

With booming NGS applications, speed has becomes a key demand. The first-generation FANSe algorithm was slow and single-threaded. FANSe2 introduced the parallelization feature utilizing multi-core central processing units (CPUs). However, the parallelization efficiency of FANSe2 limits the performance gain when more than eight CPU cores are present in the system. In addition, the insertion and deletion (indel) in FANSe2 is very time-consuming, restricting its application in precision medicine, where indel detection from WGS or WES data is routinely used. With the increase of CPU core numbers of modern servers and the development of cloud-computing infrastructures, we aimed to implement computational improvements to solve the aforementioned problems to achieve a higher mapping speed without compromising accuracy.

## Materials and Methods

### Server Configurations

As FANSe3 was designed exclusively for cloud-computing infrastructures, all tests were conducted on six server nodes using the hardware configurations listed in Table 1. A solid-state drive was installed on these server nodes to maximize the I/O throughput.

**Table 1:** Server nodes used in this study

CPU	Cores/threads	RAM
Dual Intel Xeon X5650	12C/24T	96 GB DDR3 ECC REG
Dual Intel Xeon E5-2670	16C/32T	128 GB DDR3 ECC REG
Dual Intel Xeon E5-2680V2	20C/40T	128 GB DDR3 ECC REG
Dual Intel Xeon E5-2696V4	44C/88T	256 GB DDR4 ECC REG
Quad Intel Xeon E7-4890V2	60C/120T	256 GB DDR3 ECC REG
Single AMD Threadripper 3990X	64C/128T	256 GB DDR4

### WGS Datasets

A WGS dataset generated using a BGISEQ-500 sequencer (2 × 100 bp) was downloaded from the European Nucleotide Archive (accession no.: ERP021460). An Illumina HiSeq X Ten (2 × 150 bp) WGS datase was downloaded from the Sequence Read Archive (SRA) database (accession no: SRR6656084).

### WES Dataset

A WES dataset of a tumor tissue from a lung adenocarcinoma (LUAD) patient was downloaded from the SRA database (accession no.: SRR6656083). In brief, exome capture was performed using the Agilent SureSelect Human All Exon V4 kit. Sequencing was performed on an Illumina HiSeq X Ten sequencer in the 2 × 150 bp mode.

For variant calling (SNVs and indels), as the FANSe algorithms do not export results in the BAM/SAM format, we developed a variant calling procedure using simple read pile-up and Fisher’s exact test reported previously (Wu 2014). This variant calling procedure has been experimentally validated based on nearly 2000 sites, without false positives and false negatives. In this study, we used a highly parallelized implementation of such an algorithm running in Chi-Cloud for variant calling.

### RNA-seq Datasets

Published RNA-seq datasets of A549 LUAD cells generated using Illumina and Ion torrent Proton sequencers (Mai 2017; Wang 2013) were downloaded from the SRA database (accessions nos: SRR611119 and SRR4346573).

## Results

### Improvements of FANSe3

The FANSe series of algorithms are seed-hash algorithms (like BLAST), which divide a read into several seeds. The algorithm search for perfect matches of these seeds in the reference sequences to form hotspots, which are then merged to prioritize the most probable mapping positions. To speed up the seed search, seed-hash algorithms usually implement indexing of the reference sequences. However, one of the major drawbacks is that the genome index requires an enormous amount of RAM that is proportional to the length of the reference sequence. To reduce RAM usage for normal desktop computers, FANSe2 splits the genome into small segments. However, smaller segments imply a lower probability that a read can be mapped with a particular segment. Therefore, to exclude a read that cannot be mapped with a particular segment, FANSe2 has to check all possible hotspots, i.e., to perform Smith–Waterman alignmenst for all hotspots, which is very slow. As, FANSe3 was designed for cloud-computing servers, the index can be built for the entire genome to ensure that the global high-score hotspot is always prioritized. However, this leads to high RAM usage, and 64 GB RAM is required for the mapping of reads to a human genome in a multi-core system. This is limiting factor for regular personal computers but is fully practical for cloud computing or supercomputing infrastructures. Hovever, this offers a significant advantage over FANSe2 and allows 8–10-fold increase in speed.

Due to the increase in read length and accuracy of the modern sequencers, the “step-down” of the seed length in FANSe2, i.e., to first map the reads with large seeds and then map the unmapped reads with shorter seeds, is not efficient in most cases. Therefore, FANSe3 instead of a single seed length (which can be set from 6 to 14 with an increment of 1) was used throughout the mapping process. To accelerate indel detection, fuzzy indexing was used, so that short indels are neglected during hotspot generation and are identified in the final check process. This results in an approximately 10% performance decrease when indel detection is switched on, which is substantially better than that of more than 80% speed loss in FANSe2. Additionally, the multimapping process is strongly improved in FANSe3. The Multimapped reads can be exported to a separate output file, leaving the uniquely mapped reads in one file to facilitate downstream analyses that use only these reads. Going through all possible multimapping hotspots is a step in the processing of every read. A quick multimapping estimation is performed during hotspot merging. Therefore, exporting all best mapping locations does not require extra processing time.

Extreme caution was taken to avoid thread contention when coding FANSe3. Thread-level parallelization was used in FANSe3 instead of the process-level FANSe2. This change prevents FANSe3 from parallelizing across multiple computers/server nodes. However, FANSe3 runs extremely fast in a multi-core server node and does not need to collaborate with other nodes practically. The advantage of this change lays in the fact that FANSe3 does not need an MPICH2 environment, which is often problematic during installation and configuration.

Besides the aforementioned algorithmic improvements, numerous technical improvements were made to achieve a 30–50 × fold increase in speed when mapping WGS/WES data. For example, memory accession modes were optimized specifically for the dual ring bus structure of Intel Xeon E5-V2/V3/V4 CPUs and the mesh structures of Intel Xeon Scalable/AMD Epyc CPUs. In these CPU structures, massive creation and destruction of objects, such as arrays and structures, would make other cores wait for this operation and thus decrease the overall performance. The use of more cores would exacerbate this problem. Therefore, we assigned static memory to each core and minimized array creation in the code. This approach allows a 10–20 -fold increase in speed. As indel detection is often needed, we wrote the Smith–Waterman alignment procedures before the main program, not as a function to call, to avoid an additional cost of calling functions and returning results as structures. This saves 10 μs per read at least in our computers. Considering that the mapping speed may exceed 30 million reads/min, which is equivalent to 2 μs/read, this coding trick allows an at least 5-fold increase in speed.

In summary, FANSe3 is designed for extreme speed to run in a multi-core server node with large RAM. Like in FANSe2, accuracy is still guaranteed by the mathematical estimation that ensures the mismapping rate of <10^-^^9^ (Xiao 2014).

### FANSe3 Maps 30× Human WGS Data Within 30 Minutes

**Fig. 1:**
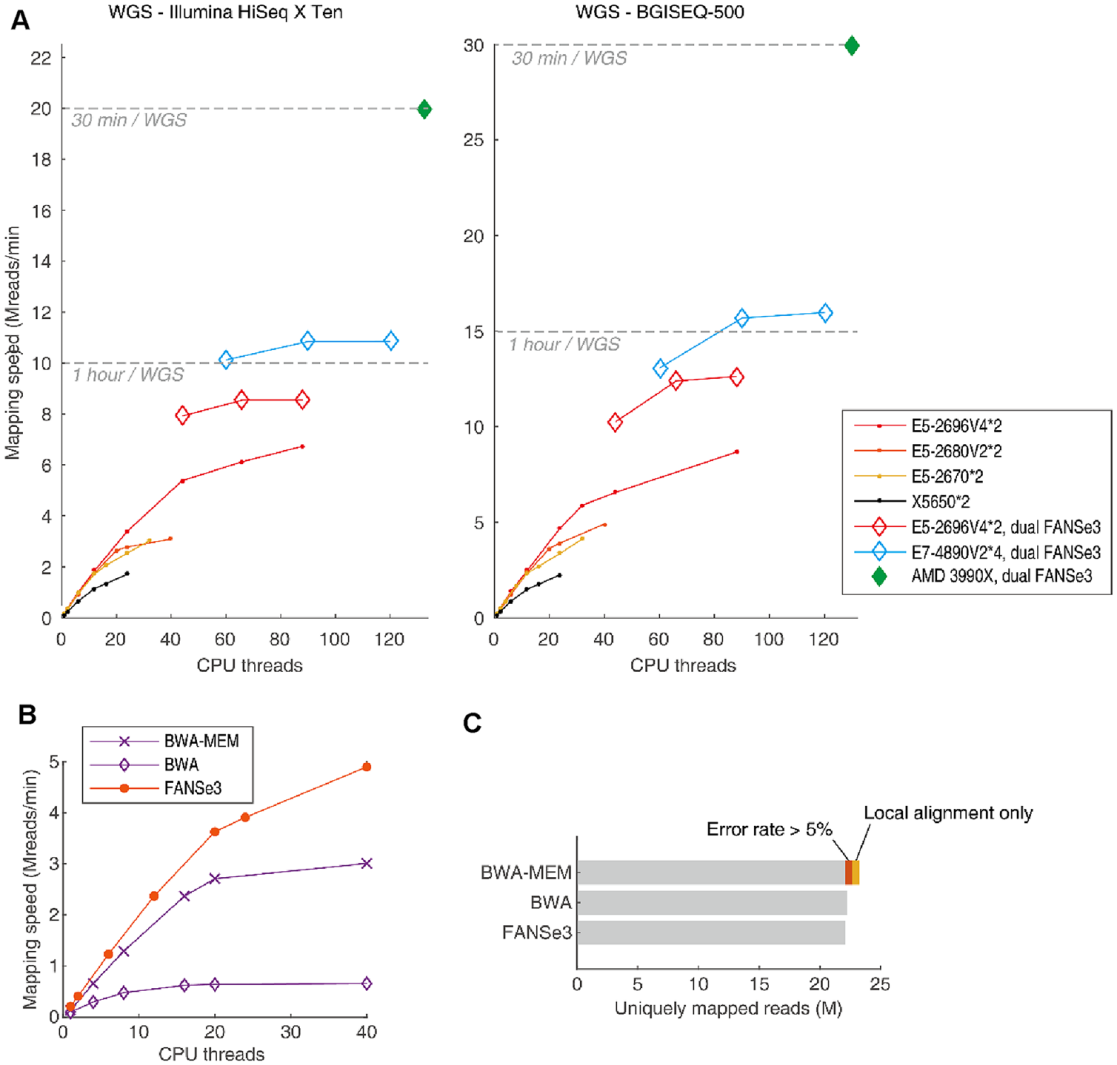
Mapping human WGS datasets against the human reference genome GRCh37/hg19. **a** Open diamonds represent the total mapping speed when running two FANSe3 processes to map reads of both ends simultaneously in one server, and dots represent the speed when running a single FANSe3 process. Dashed gray lines indicate the minimum mapping speed required to map a 30× human WGS dataset within 1 h or 30 min. **b** Mapping of the BGISEQ-500 WGS dataset using BWA, BWA-MEM, and FANSe3 on the dual E5-2680V2 system. BWA and BWA-MEM were run using default settings. **c** Uniquely mapped reads of the dataset ERR1831354 (file1, 100 nt read length) exported by BWA, BWA-MEM, and FANSe3. “Local alignment only” indicates that only part of the reads was aligned to the reference sequence. BWA and FANSe3 in this test allow a maximum of five errors

Population-scale WGS/WES puts an enormous burden on data processing capacity. We mapped WGS datasets sequenced using Illumina HiSeq X Ten (150-nt paired-end reads) and BGISEQ-500 (100-nt paired-end reads) sequencers, as these are two representative and practical WGS-scale sequencers. When running a single FANSe3 process on the six servers listed in Table 1, Intel Xeon E5 CPUs with the ring bus across four generations (E5-2670, E5-2680V2, E5-2696V4) achieved nearly identical speeds when using 12 CPU cores or less and were significantly faster than the Xeon X5650 CPU which lacks the ring bus (Fig. 1a), demonstrating that the FANSe3 algorithm was optimized for the ring bus of modern multi-core CPUs. The mapping speed increased almost proportionally to the thread count before the physical CPU core count were achieved, showing that the parallelization efficiency was optimized and FANSe3 takes full advantage of the booming cores of newer generation CPUs. When setting more threads, the performance further increased by ~ 25%, indicating that FANSe3 takes advantage of the hyper-threading feature of these modern CPUs.

Although mapping more than 6 million reads per minute is already quite fast, we noticed that reading the dataset took considerable time, during which the CPU was hardly used. To make full use of the CPU power, we ran two FANSe3 processes to map both ends of the reads simultaneously on the same server with 256 GB RAM. As expected, the speed increased by 25–30% (Fig. 1a, open diamonds). With this trick, a 6-year-old quad Xeon E7-4890V2 system with 60 CPU cores could map a 30 × human WGS dataset within 55 min, and modern AMD Threadripper 3990X CPU could complete this task in approximately 30 min (Fig. 1a, green diamond). For the BGISEQ-500 sequencer with slightly higher error rate and shorter read length, FANSe3 achieved a similar performance. We believe that the up-to-date dual Intel Xeon Scalable and dual AMD Epyc CPU systems would be even faster as they possess more cores and a mesh bus with decreased latency.

Compared to the up-to-date BWA (v. 0.7.17) running on CentOS 7 and on the Dual E5-2680V2 hardware system, FANSe3 was 60% faster than BWA-MEM and 7.5 times faster than BWA running at normal mode (Fig. 1b). Notably, FANSe3 achieved an additional 35% increase in speed when using hyper-threading, whereas BWA running on both modes achieved little performance gain by hyper-threading. Running two BWA instances simultaneously would not improve speed. This again showed the advantage of FANSe3 in terms of scalability. The mapping results of BWA and FANSe3 were nearly identical. The only exception was that BWA-MEM mapped 5.2% more reads than the other two algorithms (Fig. 1c). However, BWA-MEM performs only local alignments and does not allow the user to set a maximum number of mismatches. When we excluded the “local-alignment-only” reads and reads with an error rate higher than 5%, BWA-MEM yielded a nearly identical number of uniquely mapped reads like BWA and FANSe3.

### FANSe3 Maps WES Datasets Robustly Within Minutes

**Fig. 2:**
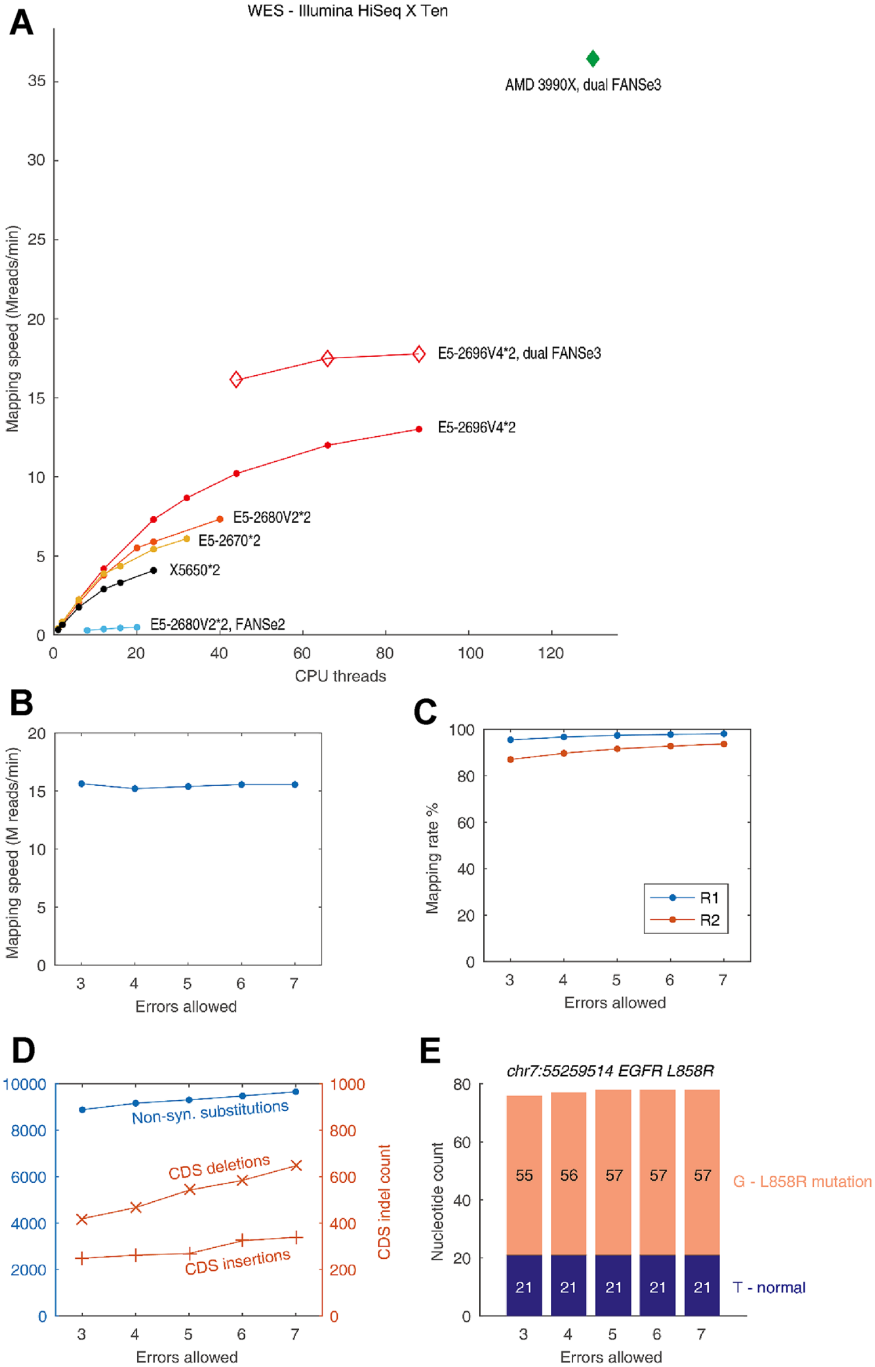
WES data analysis using FANSe3. **a** Speed test of FANSe3 in various systems with five errors allowed and indel detection on. The blue graph indicate the speed of FANSe2 mapping for the same dataset with indel detection off and masked genome on. **b** Mapping speed of FANSe3 allowing 3–7 errors per read. **c** Mapping rates of both ends of the paired-end reads at different error allowances. **d** Detection of non-synonymous substitutions and coding-sequence indels at different error allowances. **e** Nucleotide pile-ups at chr7:55259514 to detect T ≥ G mutation (causing EGFR L858R driver mutation)

WES is often used in clinical applications to sequence disease-relevant mutations at a substantially higher depth than that of WGS at a reasonable cost. In this study, we used WES data of a tumor tissue from an LUAD patient to search for druggable driver mutations. This dataset contained 41.5 M paired-end reads, with a nominal sequencing depth of 244× on average. Since exons are rarely repetitive sequences, FANSe3 could map these reads more efficiently than WGS reads to the reference genome, achieving nearly doubled mapping speed compared to that for the WGS datasets (Fig. 2a). As observed for the WGS data, running two FANSe3 processes to map both ends simultaneously utilized the CPU power with higher efficiency. A dual Xeon E5-2696V4 system achieved a speed of 17.78 M reads/min, indicating that a 50× WES dataset (~ 2.5 GB data, generally used for genetic testing and genome-wide association studies) could be mapped in approximately 30 s (Fig. 2a).

For clinical applications, robustness is essential. We compared the mapping results when allowing 3–7 errors per read. Increasing the error allowance did not substantially influence the mapping speed (Fig. 2b). The mapping rate of the first end remained nearly constant when the error allowance increased. Due to the lower sequencing quality, the mapping rate of the second end was lower, and it increased slightly with the increase of error allowance (Fig. 2c). This led to a slight increase in detected non-synonymous substitutions and indels (Fig. 2d). Nevertheless, at the position of clinical significance, the T > G variant causing EGFR L858R mutation, displayed almost the same nucleotide pile-up result, which is independent of parameter settings (Fig. 2e). This finding was validated by qPCR. Based on these findings, the patient was treated with erlotinib, a tyrosine kinase inhibitor targeting the EGFR L858R mutation, and the tumor decreased considerably. The treatment outcome validated the robust findings from the WES data, demonstrating its clinical potential.

### FANSe3 Maps RNA-Seq Reads with higher speed

**Fig. 3:**
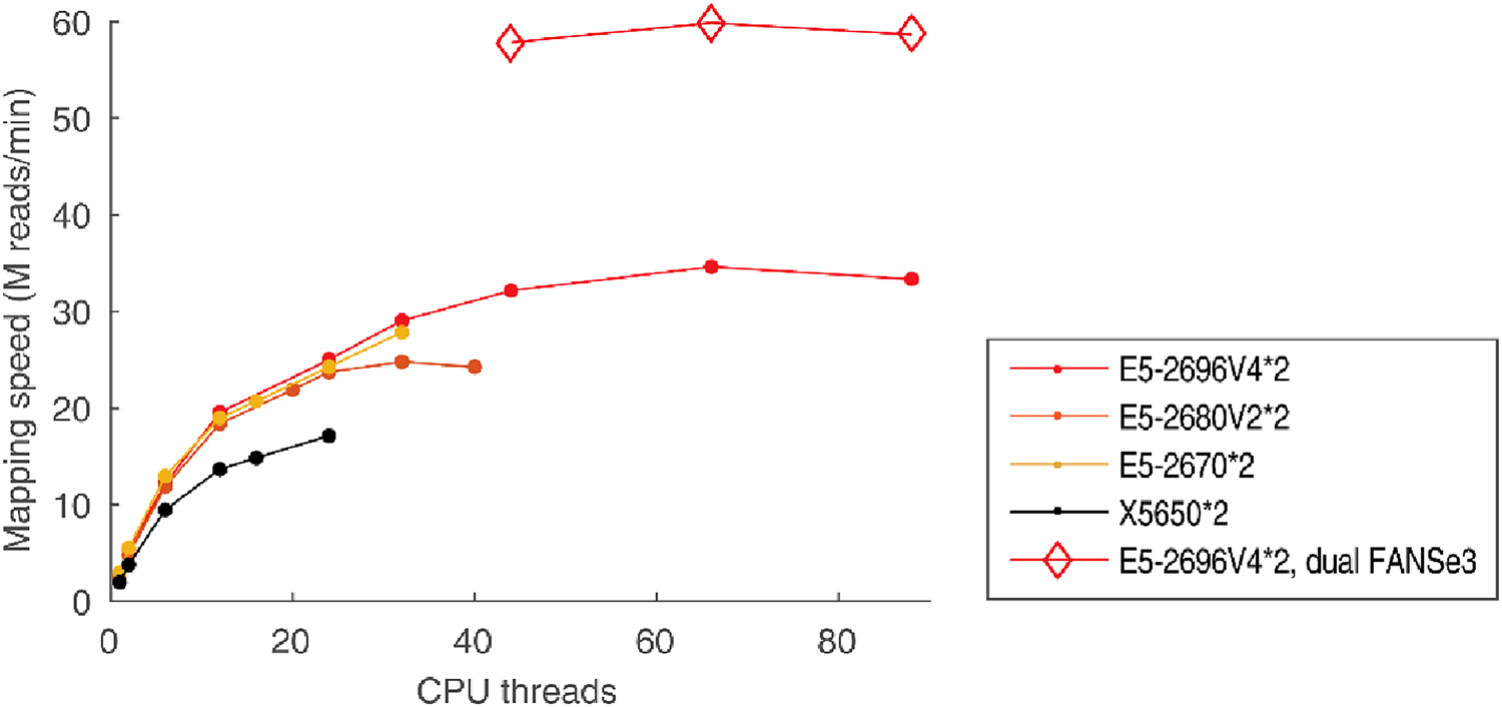
Mapping an A549 cell mRNA-seq dataset to NCBI RefSeq-RNA reference sequences using FANSe3 on various platforms

Like its previous generations, FANSe3 does not support the spliced mapping directly for RNA-seq, as FANSe2splice (Mai 2017) is specifically designed for this purpose. In most RNA-seq applications, where novel splice junctions do not need to be discovered, directly mapping reads to already spliced RNA reference sequences is a substantially faster and more accurate solution. We previously reported that FANSe3 is at least 30 times faster than FANSe2 in RNA-seq applications (Liu 2018). We mapped a previously generated mRNA-seq dataset of A549 LUAD cells (75-nt single-ended reads) (Wang 2013) to NCBI RefSeq-RNA reference sequences. Given the significantly smaller reference sequences, the computational complexity is substantially lower than that in the WGS/WES applications. Therefore, disk I/O becomes a bottleneck. In the test, the parallelization efficiency dropped when more than 12 cores were enabled (Fig. 3). Dual FANSe3 processes utilized the CPU more efficiently and achieved a mapping speed of 59.9 M reads/min or almost 1 M reads/s. At this speed, a typical mRNA-seq dataset, which is sufficient to quantify more than 11,000 genes in human cells (2 M reads, Human Proteome Project) (Chang 2014), could be accomplished in only 2 s. In addition, even running two FANSe3 processes simultaneously used only approximately half of the CPU power of the dual Xeon E5-2696V4 system because of the insufficient I/O speed of the SSD. We believe that using a substantially faster enterprise-level storage system may unleash the computational power to achieve 1 s per mRNA-seq dataset in a single server node.

### Parameter Robustness for RNA Quantification Using FANSe3

Given the extra steps in RNA-seq library construction compared to DNA-seq library construction, e.g., reverse transcription, the error rate of RNA-seq is generally higher than that of DNA-seq. Lower-throughput and error-prone sequencers such as the Ion Torrent are often used for transcriptome sequencing. Therefore, mapping algorithms must deal with higher and uncertain error rates. This requires robustness of the mapping algorithms. Thus, we tested the parameter robustness of FANSe3 using A549 cell RNA-seq data generated by Illumina and Ion Torrent sequencers.

**Fig. 4:**
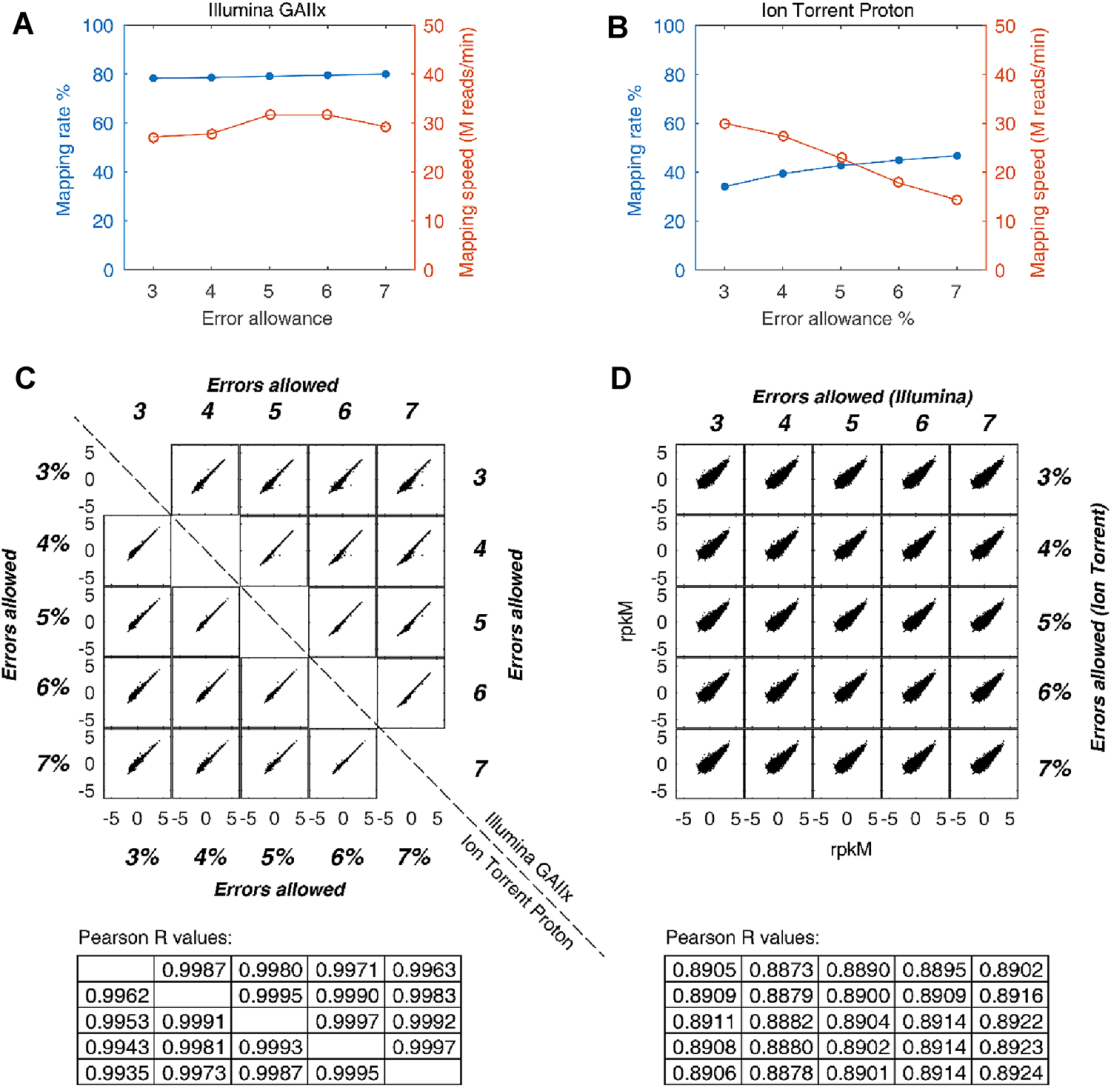
Parameter robustness of FANSe3 in RNA-seq applications. A549 cell mRNA-seq data generated using Illumina GAIIx and Ion Torrent Proton sequencers were mapped to RefSeq-RNA reference sequences. **a**, **b** Mapping rate and mapping speed when allowing 3–7 errors for the Illumina dataset and 3–7% errors for the Ion Torrent Proton dataset (due to the variable read length of the Ion Torrent sequencer). Mapping speed was recorded using a single FANSe process in the dual E5-2696V4 server node. **c** Correlation of RNA quantification (in RPKM) when setting different error allowances for the Illumina and Ion Torrent datasets. **d** Correlation of RNA quantification among the sequencing platforms under different error allowances

For the Illumina dataset, allowing 3–7 errors yielded very similar mapping rates, and the mapping speed remained quite constant (Fig. 4a). For the Ion Torrent Proton dataset, the mapping rate was generally higher than that of the Illumina dataset owing to the higher error rate. With the increasing error allowance from 3% to 7%, the mapping rate increased from 34.1% to 46.7% at the cost of decreased mapping speed (Fig. 4b). Interestingly, the quantification of gene expression was nearly independent from the error allowance: the Pearson *R* values for the same sequencer were all above 0.993 (Fig. 4c). Correlation coefficients for any mRNA quantified by the two sequencers were nearly all around 0.89, suggesting it is independent from the error allowance (Fig. 4d). These results demonstrated the parameter robustness of FANSe3 in RNA-seq applications, and the results are largely independent of parameter settings.

## Discussion

With 30 min for WGS data, 1 min for WES data, and 1–2 s for transcriptome data, FANSe3 sets a new speed record for NGS analysis. This speed was fully achieved on CPU and was even substantially higher than that of many FPGA/GPU solutions, demonstrating the advantageous algorithm design. FANSe3 does not use any special instruction sets, such as SSE and AVX, indicating that it can be theoretically compiled to run on any platform even non-X86 systems. This versatility allows convenient deployment in sequencing centers to utilize the power of national supercomputing centers. For example, FANSe3 deployed in the TianHe-2 supercomputer (Guangzhou, China) would be able to map 50 × WES datasets of the entire world population (7.8 billion people) within 1 year. This speed may facilitate a boom in rapid clinical applications (e.g., pathogen identification) and population-scale research/applications. Moreover, this performance also dramatically decreases the computational cost of NGS, facilitating low-cost NGS applications that are widely affordable.

With the almost identical mapping results to those with FANSe2 (Liu 2018), FANSe3 inherited its the extreme accuracy and robustness. We demonstrated the robustness in both DNA-seq and RNA-seq applications, where SNV identification and RNA quantification were independent of the parameter settings, providing a potential solution to the “alarming reproducibility crisis” (Nekrutenko and Taylor 2012). This is essential for the clinical use, and our algorithm provides a strong candidate for a standardized NGS workflow procedure.

The major drawback of FANSe3 is the high RAM demand. Indeed, FANSe3 is designed exclusively for high-performance computing centers and cloud-computing infrastructure. It can be run on regular personal computers only when small reference sequences are used, e.g., bacterial genomes. Another drawback is I/O bottleneck in the WES and transcriptome applications, where the CPU maps the reads faster than the hard disk can read/write (Figs. 2, 3). This depends on the hardware development. In a cloud-computing environment, the user experience may also be limited by slow network transfer for the extremely large sequencing datasets. However, with the booming broad-band network and 5G wireless communication technology, everyone can upload compressed sequencing datasets to the cloud and obtain analysis results quickly without the local computational resources. This will dramatically promote NGS applications in various fields, including mobile medical services, on-site forensic interrogations and environmental monitoring.
